# Supplementary material for: Psychometric Validation of the Arabic Version of the WPAI:Migraine Questionnaire in Patients with Migraine
Source: Neurol Int. 2025 Dec 12;17(12):202. doi: 10.3390/neurolint17120202 (PMC12736024; doi:10.3390/neurolint17120202)
Supplement: Supplementary file 1 [file neurolint-17-00202-s001.zip › neurolint-3992904-supplementary.pdf]

استبيان الإنتاجية في العمل وإعاقة النشاط (WPAI)

تدور الأسئلة التالية حول تأثير الصداع النصفي (الشقيقة) على قدرتك على العمل والقيام بأنشطتك المعتادة. يرجى ملء الفراغات أو وضع دائرة حول الرقم المناسب بحسب ما هو موضح:

١- هل تشغل وظيفة ما في الوقت الحالي (تتلقى أجرا لقاء عملك)؟ ◯نعم ◯لا

إذا كانت الإجابة لا، انتقل مباشرة إلى السؤال رقم ٦

٢ -في خلال الأيام السبعة الأخيرة، كم بلغ عدد الساعات التي تغيبت فيها عن العمل بسبب المشاكل المرتبطة بالصداع النصفي؟

قم بإضافة إجمالي الساعات التي تغيبت فيها بسبب الإجازات المرضية والأوقات التي وصلت فيها متأخرا أو غادرت فيه مبكرا، وما إلى ذلك، بسبب الصداع النصفي. لا تقم بتضمين الفترة الزمنية التي تغيبت فيها للمشاركة في هذه الدراسة

.. ساعة (ساعات)

٣- في خلال الأيام السبعة الأخيرة، كم بلغ عدد الساعات التي تغيبت فيها عن العمل لأي سبب آخر، مثل عطلة أو إجازة أو التغيب للمشاركة في هذه الدراسة؟

.. ساعة (ساعات)

٤- في خلال الأيام السبعة الماضية، كم بلغ عدد ساعات عملك الفعلية؟

.. ساعة (ساعات) (إذا كانت الإجابة صفر فانتقل إلى السؤال رقم ٦)

٥ -في خلال الأيام السبعة الماضية، ما مدى تأثير الصداع النصفي على معدل إنتاجيتك أثناء عملك؟

فكر في الأيام التي كنت فيها مضطرا للتقصير من حيث كمية أو نوع العمل الذي يمكنك القيام به أو نوعه والأيام التي أنجزت فيها قدرا أقل مما كنت ترغب فيه أو الأيام التي لم تستطع فيها القيام بعملك بعناية كالمعتاد. إذا كان الصداع النصفي قد أثر على عملك بشكل بسيط، فاختر رقما منخفضا. واختر رقما مرتفعا إذا كان الصداع النصفي قد أثر على عملك بشكل كبير. ضع دائرة حول الرقم المناسب.

لم يؤثر الصداع النصفي على عملي ← ١ ٢ ٣ ٤ ٥ ٦ ٧ ٨ ٩ ١٠ → منعني الصداع النصفي تماما عن العمل

٦ -في خلال الأيام السبعة الماضية، ما مدى تأثير الصداع النصفي على قدرتك على القيام بأنشطتك اليومية المعتادة، بخلاف أداء وظيفتك؟

المقصود بالأنشطة المعتادة الأنشطة الاعتيادية التي تقوم بها كالعمل حول المنزل أو التسوق أو رعاية الأطفال أو ممارسة التمرينات الرياضية أو الدراسة، وإلى ما هنالك. فكر في الأوقات التي كنت فيها مقيدا بنوع وحجم معين من الأنشطة التي يمكنك القيام بها والأوقات التي أنجزت فيها أقل مما ترغب. إذا كان الصداع النصفي قد أثر على أنشطتك بشكل بسيط، فاختر رقما منخفضا. واختر رقما مرتفعا إذا كان الصداع النصفي قد أثر على أنشطتك بشكل كبير. ضع دائرة حول الرقم المناسب.

لم يؤثر الصداع النصفي على أنشطتي اليومية ← ١ ٢ ٣ ٤ ٥ ٦ ٧ ٨ ٩ ١٠ → منعني الصداع النصفي تماما عن القيام بأنشطتي اليومية

Work Productivity and Activity Impairment Questionnaire: Migraine

The following questions ask about the effect of your MIGRAINE on your ability to work and perform regular activities. Please fill in the blanks or circle a number, as indicated.

1- Are you currently employed (working for pay)? **Yes** ◯ **No** ◯

If NO, check "NO" and skip to question 6.

2- During the past seven days, how many hours did you miss from work because of problems associated with your MIGRAINE? Include hours you missed on sick days, times you went in late, left early, etc., because of your MIGRAINE. Do not include time you missed to participate in this study.

____ HOURS

3- During the past seven days, how many hours did you miss from work because of any other reason, such as vacation, holidays, time off to participate in this study?

____HOURS

4- During the past seven days, how many hours did you actually work?

____HOURS (If "0", skip to question 6.)

5- During the past seven days, how much did your MIGRAINE affect your productivity while you were working?

Think about days you were limited in the amount or kind of work you could do, days you accomplished less than you would like, or days you could not do your work as carefully as usual. If MIGRAINE affected your work only a little, choose a low number. Choose a high number if MIGRAINE affected your work a great deal.

Consider only how much MIGRAINE affected productivity while you were working.

MIGRAINE had no effect on my

1 2 3 4 5 6 7 8 9 10

MIGRAINE Completely

6- During the past seven days, how much did your MIGRAINE affect your ability to do your regular daily activities, other than work at a job?

By regular activities, we mean the usual activities you do, such as work around the house, shopping, childcare, exercising, studying, etc. Think about times you were limited in the amount or kind of activities you could do and times you accomplished less than you would like. If MIGRAINE affected your activities only a little, choose a low number. Choose a high number if MIGRAINE affected your activities a great deal.

Consider only how much MIGRAINE affected your ability to do your regular daily activities, other than work at a job.

MIGRAINE had no effect on my daily activities

1 2 3 4 5 6 7 8 9 10

MIGRAINE completely prevented me from doing my daily activities
